# Acute and long-term outcomes of ICU-acquired weakness: a cohort study and propensity matched analysis

**DOI:** 10.1186/cc13656

**Published:** 2014-03-17

**Authors:** G Hermans, H Van Mechelen, B Clerckx, T Vanhullenbusch, D Mesotten, A Wilmer, MP Casaer, P Meersseman, Y Debaveye, PJ Wouters, R Gosselink, G Van den Berghe

**Affiliations:** 1 UZ Leuven, Belgium; 2 KU Leuven, Belgium

## Introduction

ICU-acquired weakness is a frequent complication of critical illness. It is unclear whether it is a marker or mediator of poor outcomes. We aimed to determine acute and long-term outcomes and costs of ICU-acquired weakness among long-stay (≥8 days) ICU patients and to assess the impact of recovery of weakness at ICU discharge.

## Methods

Data were prospectively collected during an RCT (http://Clinicaltrials.gov: NCT00512122) [1,2]. Impact of weakness (MRC sum <48) on outcomes and costs was analyzed with 1:1 propensity score-matching for baseline characteristics, illness severity and risk factor exposure prior to assessment. Among weak patients, impact of persisting weakness at ICU discharge on risk of death after 1 year was examined with multivariable Cox proportional-hazard analysis.

## Results

A total 227 of the 405 (56%) long-stay assessable ICU patients were weak; 122 weak patients were matched to 122 not-weak patients. As compared with matched not-weak patients, weak patients had a lower likelihood at any time for live weaning from mechanical ventilation (HR: 0.709 (0.548 to 0.918), *P *= 0.009), live ICU (HR: 0.738 (0.570 to 0.955), *P *= 0.021) and hospital discharge (HR: 0.682 (0.521 to 0.893), *P *= 0.005). In-hospital costs/patient (+30.5%, €+5,443/patient, *P *= 0.04) and 1-year mortality (30.6% vs. 17.2%, *P *= 0.015) were also higher. The 105/227 (46%) weak patients not matchable to not-weak patients had even worse prognosis and higher costs. At any time within the first year following ICU admission, compared with patients who recovered from weakness and adjusted for potential confounders, those with persistent weakness and MRC sum between 36 and 47 at ICU discharge had a higher likelihood of death (HR: 1.937, 95% CI: 1.048 to 3.581, *P *= 0.035). This likelihood of late death was even higher for those patients with a more severe degree of persistent weakness (MRC sum <36) (HR: 1.815, 95% CI: 3.693 to 7.514, *P *< 0.001).

## Conclusion

Patients with ICU-acquired weakness had worse acute morbidity outcomes, consumed more resources and revealed higher mortality after 1 year than patients without weakness. Persistence of weakness at ICU discharge further increased late mortality. **Acknowledgement **GH and HVM contributed equally to this study.
